# Planning for Seniors Housing in Changing Cities: Lessons Learned From a Cross-National Exchange

**DOI:** 10.1093/geroni/igab046.1459

**Published:** 2021-12-17

**Authors:** Christine Sheppard, Tam Perry, Karen Kobayashi, Denise Cloutier, Yasir Mehmood, Emma Helfand-Green, Sander Hitzig

**Affiliations:** 1 Sunnybrook Research Institute, Toronto, Ontario, Canada; 2 Wayne State University, Detroit, Michigan, United States; 3 University of Victoria, Victoria, British Columbia, Canada; 4 Wayne State University, Wayne State, Michigan, United States; 5 City of Toronto, Toronto, Ontario, Canada

## Abstract

Across North America, a growing number of older adults have a core housing need and lack access to affordable, suitable or adequate housing. Although federal, state/provincial and local backdrops vary across Canadian and American contexts, seniors’ housing providers in both countries face similar challenges and must develop innovative policy and program responses to help older adults age in place. We hosted an international seniors’ housing conference to create a platform for cross-national collaboration among multidisciplinary seniors housing experts. This event offered an opportunity to exchange best practices, emerging research, and policy solutions, and establish a set of shared priorities for advancing seniors housing that were applicable to two nations with different social systems. This paper will reflect on the exchange of knowledge and best practices related to housing preservation, eviction prevention, and access to supports during COVID-19, and the lessons learned fostering a cross-national collaborative network of seniors housing experts.

